# Supplementary material for: Cessation of Face Mask Use after COVID-19 Vaccination in Patients with Diabetes: Prevalence and Determinants
Source: Int J Environ Res Public Health. 2023 Feb 4;20(4):2768. doi: 10.3390/ijerph20042768 (PMC9956089; doi:10.3390/ijerph20042768)
Supplement: Supplementary file 1 [file ijerph-20-02768-s001.zip › ijerph-2186317-supplementary.pdf]

Supplementary File S1. QUESTIONNAIRE

FILTER QUESTION: Have you already received at least one dose of the coronavirus vaccine? **Answer: Yes** (invite the patient to participate in the study). **No** (thank the patient for responding and find another participant).

Response date (dd/mm/yyyy): ___/_____/_____.

Section 1. Use of face mask before and after COVID-19 vaccination

Before you were vaccinated against COVID-19, how often did you wear a face mask in the following situations?

1. Inside the car/bus/subway:

1. Never	2. Rarely	3. Sometimes	4. Almost always	5. Always

2. Walking outdoors:

1. Never	2. Rarely	3. Sometimes	4. Almost always	5. Always

3. Talking inside the house:

1. Never	2. Rarely	3. Sometimes	4. Almost always	5. Always

4. Talking outside the house:

1. Never	2. Rarely	3. Sometimes	4. Almost always	5. Always

5. Waiting for food in a restaurant:

1. Never	2. Rarely	3. Sometimes	4. Almost always	5. Always

After you were vaccinated against COVID-19, how often do you wear a face mask in the following situations?

6. Inside the car/bus/subway:

1. Never	2. Rarely	3. Sometimes	4. Almost always	5. Always

7. Walking outdoors:

1. Never	2. Rarely	3. Sometimes	4. Almost always	5. Always

8. Talking inside the house:

1. Never	2. Rarely	3. Sometimes	4. Almost always	5. Always

9. Talking outside the house:

1. Never	2. Rarely	3. Sometimes	4. Almost always	5. Always

10. Waiting for food in a restaurant:

1. Never	2. Rarely	3. Sometimes	4. Almost always	5. Always

Section 2. Perceived vulnerability to COVID-19 before and after vaccination

Before you were vaccinated against COVID-19…

11. How vulnerable you felt you would get sick from COVID?

1. Not any	2. Very low	3. Low	4. High	5. Very high

12. How vulnerable you felt you would have symptoms of COVID?

1. Not any	2. Very low	3. Low	4. High	5. Very high

13. How vulnerable you felt you would need hospitalization because of COVID?

1. Not any	2. Very low	3. Low	4. High	5. Very high

14. How vulnerable you felt you would die from COVID?

1. Not any	2. Very low	3. Low	4. High	5. Very high

Section 3. Benefits of wearing masks. Please choose as honestly as possible the option that most applies to you:

15. The mask is useful, with or without symptoms of COVID-19

-1. No	0. No	1. Yes

16. The mask avoids infecting loved ones

-1. No	0. No	1. Yes

17. People around you can feel safe if you wear a mask

-1. No	0. No	1. Yes

18. The mask protects from infecting others

-1. No	0. No	1. Yes

19. The mask helps control the coronavirus pandemic

-1. No	0. No	1. Yes

20. The mask protects from getting COVID-19

-1. No	0. No	1. Yes

21. The mask gives the peace of mind to avoid contagion

-1. No	0. No	1. Yes

22. The mask prevents getting infected with COVID-19

-1. No	0. No	1. Yes

23. The mask protects me from other diseases

-1. No	0. No	1. Yes

24. The mask keeps you safe from coronavirus

-1. No	0. No	1. Yes

Section 4. Barriers to wearing masks. Please choose as honestly as possible the option that most applies to you:

25. With the hot weather it is difficult to maintain the use of the mask

-1. No	0. No	1. Yes

26. Wearing the mask is bothersome

-1. No	0. No	1. Yes

27. Wearing the mask is uncomfortable

-1. No	0. No	1. Yes

28. With the mask on, it's hard to breathe

-1. No	0. No	1. Yes

29. With the mask on, it is difficult for me to speak

-1. No	0. No	1. Yes

30. Buying masks affects my economy

-1. No	0. No	1. Yes

31. The mask causes skin problems

-1. No	0. No	1. Yes

32. The mask is unnecessary if you are already vaccinated

-1. No	0. No	1. Yes

33. The mask is only useful for people who have symptoms

-1. No	0. No	1. Yes

34. The mask is unnecessary if you already got COVID-19

-1. No	0. No	1. Yes

Section 5. Self-efficacy of wearing masks

How capable do you feel to use the mask if you were in the following situations?

35. While being inside of a crowded place

1. Not any	2. Very low	3. Low	4. High	5. Very high

36. Despite there are people who think face masks are useless

1. Not any	2. Very low	3. Low	4. High	5. Very high

37. Despite keeping it on is annoying

1. Not any	2. Very low	3. Low	4. High	5. Very high

38. Despite it is difficult to breathe with the mask on

1. Not any	2. Very low	3. Low	4. High	5. Very high

39. Despite keeping it on is uncomfortable

1. Not any	2. Very low	3. Low	4. High	5. Very high

40. While waiting for food in a restaurant

1. Not any	2. Very low	3. Low	4. High	5. Very high

41. While meeting/partying with family and friends

1. Not any	2. Very low	3. Low	4. High	5. Very high

Section 6. Vaccine´s expectations: What is your opinion of each of the following statements?

42. The vaccine reduces the risk of hospitalization for COVID-19

-1. No	0. No	1. Yes

43. The vaccine reduces the risk of dying from COVID-19

-1. No	0. No	1. Yes

44. The vaccine reduces the spread of the disease

-1. No	0. No	1. Yes

45. The vaccine totally prevents contagion

-1. No	0. No	1. Yes

46. The vaccine allows stop using the face masks

-1. No	0. No	1. Yes

47. The vaccine allows stop using antibacterial gel

-1. No	0. No	1. Yes

Section 7. Sociodemographic variables and medical history

48. Sex: Female___ Male___
49. Age: ____
50. Marital status (lives with a partner): Yes___ No___
51. Occupation (economically active): Yes___ No___
52. Schooling: High school or less: ___ Bachelor's degree or higher: ___
53. Smoking: Never: ___ I used to smoke in the past: ___ I currently smoke: ___

Medical history: Has a doctor ever told you that you have one of the following diseases?

54. Hypertension: Yes___ No___

55. Chronic obstructive pulmonary disease: Yes___ No___
56. Immunosuppression: Yes___ No___
57. Chronic kidney disease: Yes___ No___
58. Cancer
    Which vaccine did you receive?
59. Pfizer: Yes___ No___
60. Cansino: Yes___ No___
61. Astra Zeneca: Yes___ No___
62. Sputnik: Yes___ No___
63. Covaxin: Yes___ No___
64. Sinovac: Yes___ No___
65. I don't know
66. How many doses did you receive?
    One dose ___ Two doses ___ Three or more doses ___

Have you gotten sick of COVID-19?

67. Never got sick ___
68. Before the 1st vaccine dose ___
69. Before the 2nd vaccine dose ___
70. After the 2nd vaccine dose ___
71. I needed hospitalization Yes___ No___ Not applicable ___
72. Has any member of your family gotten sick of COVID-19? Yes___ No___
73. Has any member of your family died because of COVID-19? Yes___ No___

Supplementary Table S1. Cessation of the use of face masks and related variables according to the number of vaccine doses received against COVID-19

	Number of doses		
	1 dose (n = 18) (%)	2 or more doses (n = 270) (%)	p value*
Cessation of use after vaccination	7 (38.9)	66 (24.4)	0.173
Not feeling vulnerable after vaccination			
For getting the infection	6 (33.3)	99 (36.7)	1.000
For developing symptoms	6 (33.3)	108 (40.0)	0.628
For being hospitalized	8 (44.4)	156 (57.8)	0.328
For dying from the disease	9 (50.0)	138 (51.1)	1.000
Perception of benefits	16 (88.9)	241 (89.3)	1.000
Perception of barriers	3 (16.7)	70 (25.9)	0.576
High and very high self-efficacy	17 (94.4)	260 (96.3)	0.515
Vaccine's realistic expectations	13 (72.2)	220 (81.5)	0.353
Vaccine's unrealistic expectations	3 (16.7)	39 (14.4)	0.733

*Chi-square test.
